# Biologic Width Following Different Crown Lengthening Procedures: A Six-Month Follow-Up Study

**DOI:** 10.7759/cureus.59325

**Published:** 2024-04-29

**Authors:** Chanchal Katariya, Arvina Rajasekar

**Affiliations:** 1 Periodontics, Saveetha Dental College and Hospitals, Saveetha Institute of Medical and Technical Sciences, Saveetha University, Chennai, IND

**Keywords:** osseous reduction, gingivectomy, crown lengthening, biologic width, apically displaced flap

## Abstract

Background: One of the important things to preserve during crown lengthening is the biologic width (BW), recently called supracrestal tissue attachment. A healthy periodontium with adequate BW is very essential for the success of restored teeth. There are various techniques to perform crown lengthening procedures. Most of the studies have focused on assessing the changes in the position of the marginal gingiva and bone as outcome parameters rather than BW. Also, most of the research was done on animal models.

Aim: The purpose of this study was to assess the periodontal tissue changes at three months and six months following two different surgical crown lengthening procedures.

Materials and methods: Sixty mandibular first molars among 60 patients that required surgical crown lengthening were enrolled in the study and subjected to two different procedures, gingivectomy (Group I; n=30) and apically positioned flap with ostectomy (Group II; n=30). The following parameters were recorded at baseline, three months, and six months, position of free gingival margin (FGM), probing depth (PD), relative attachment level (RAL), bone level (BL), and BW. These measurements were made at three sites in every patient: treated tooth sites (TT), adjacent tooth’s adjacent sites (AD), and adjacent tooth’s non-adjacent sites (NAD). The data was then subjected to statistical analysis using SPSS software (Version 20.0). Statistical significance was set to p<0.05.

Results: When groups I and II were compared at three and six months, there was no statistical difference in terms of position of FGM, PD, and RAL (p>0.05). When BW was compared between the two groups at three and six months, group II showed better reestablishment of BW at any given time period and was statistically significant (p<0.05).

Conclusion: Following surgical crown lengthening, the bone level was shifted apically and allowed for the reestablishment of BW. At six months of follow-up, the apically positioned flap with ostectomy was superior in restoring the BW compared to gingivectomy.

## Introduction

Periodontal crown lengthening is used when there is inadequate sound dental structure to establish a crown margin with proper retention and resistance. Along this process, the sustained success and positive result of a restored tooth depend on the maintenance of a healthy periodontium. Dentists must continuously strike a balance between their patients' need for aesthetics and periodontal health.

The most frequent periodontal procedure is crown lengthening, which is done to extend the teeth for better function and/or aesthetics. Crown lengthening can be used to treat short clinical crowns, caries extending subgingivally, and fractured crowns or roots. In these situations, it aids in repositioning the gingival sulcus and biological width of the supracrestal gingival tissue more apically [1,2]. This process aims to minimize the excessive gingival display and to create harmonic relations between the periodontal tissues and facial structures [3]. Gingivectomy with or without bone reduction, apically positioned flap, and gingivectomy with flapless ostectomy are some of the different techniques used for crown lengthening [4]. The selection of technique is determined by the location of the mucogingival junction, alveolar crest, gingival margin, and the potential for concomitant restorative therapy [5].

One of the important things to preserve during crown lengthening is the biologic width (BW) or called supracrestal tissue attachment. The dentogingival junction's dimension in humans was reported by Gargiulo AW et al. [6]. The junctional epithelium and connective tissue together comprise the BW [7]. Its average measurement is 2.04 mm. The average sulcus depth, epithelial attachment, and connective tissue attachment were found to be 0.69 mm, 0.97 mm, and 1.07 mm, respectively [8]. Every healthy dentition has these measurements, even though they may vary from tooth to tooth. 

It is important to maintain the biological width and not violate it. Providing restorative margins that encroach on the BW regularly causes gingival irritation and bone destruction [9]. Plaque retention and poor periodontal health are caused by restorative margin placement that is within the BW. An ongoing inflammation is caused and exacerbated by the patient's incapacity to clean this area when the restoration margin is positioned excessively below the gingival tissue crest, impinging on the gingival attachment apparatus. Studies evaluating the histological and clinical reactions of periodontium to restorative margins positioned violating the BW have supported these alterations [10-12]. In this regard, the goal of the study was to evaluate the changes in the periodontal tissue at three and six months after two distinct surgical crown lengthening procedures.

## Materials and methods

Study setting

Between January 2022 and December 2022, 60 patients with reduced crown height in the mandibular first molar who required crown lengthening were matriculated at the Department of Periodontology, Saveetha Dental College and Hospitals, Chennai, India. The institutional ethics committee accepted the study protocol (Ref- IHEC/SDC/PERIO-2001/22/057). Participants in the study gave their informed consent in all cases. The study followed the guidelines set forth in the Declaration of Helsinki, as updated in 2013. The sample size was calculated to be 60 with G*Power software, Version 3.1.9.4 (power at 80% and alpha error at 95% confidence level) by considering the mean and standard deviation values from the earlier research [13]. Systemically healthy patients belonging to physical status I based on the American Society of Anesthesiologists classification; periodontally healthy patients; patients aged between 18 and 65 years; and patients in requirement of surgical crown lengthening for marginal gingiva discrepancies, subgingival caries, short clinical crown with enough root length, subgingival restorations, or for crown placement were included in this study. Periodontally compromised teeth, systemically compromised patients, molars with furcation involvement and insufficient periodontal support, teeth with compromised crown root ratio, smokers, pregnant ladies and lactating women, and patients who had undergone periodontal therapy or any mucogingival surgery before three months were excluded from the study.

Grouping and outcome variables

Among the 60 mandibular first molar requiring surgical crown lengthening in 60 patients, 30 patients were randomly subjected to gingivectomy (Group I) and the remaining 30 patients were subjected to apically positioned flap with ostectomy (Group II).

Each patient underwent a full-mouth scaling and root planing procedure as well as the removal of any marginal irritants. Patients were summoned back for a baseline evaluation after a week of plaque control management. Each patient received a full arch acrylic stent and vertical grooves were made at the proper interproximal locations to standardize probe placement and angulation. Rounding up all measurements to the nearest millimeter was done using the UNC 15 standard probe. The variables (free gingival margin (FGM) - fixed reference point in the stent to the FGM; relative attachment level (RAL) - fixed reference point in a stent to the base of the pocket; probing depth (PD) - subtracting FGM value from RAL; bone level (BL) - after anesthetizing the site, bone sounding was done to assess the BL from the fixed reference point in stent; BW - subtracting the BL from RAL) were noted at baseline for every tooth requiring crown lengthening (treated tooth sites - TT; adjacent tooth’s adjacent sites - AD; adjacent tooth’s non-adjacent sites - NAD) in both groups at four sites (mesiobuccal, distobuccal, mesiolingual, and distolingual) and the average was recorded.

Surgical intervention

**Figure 1:**
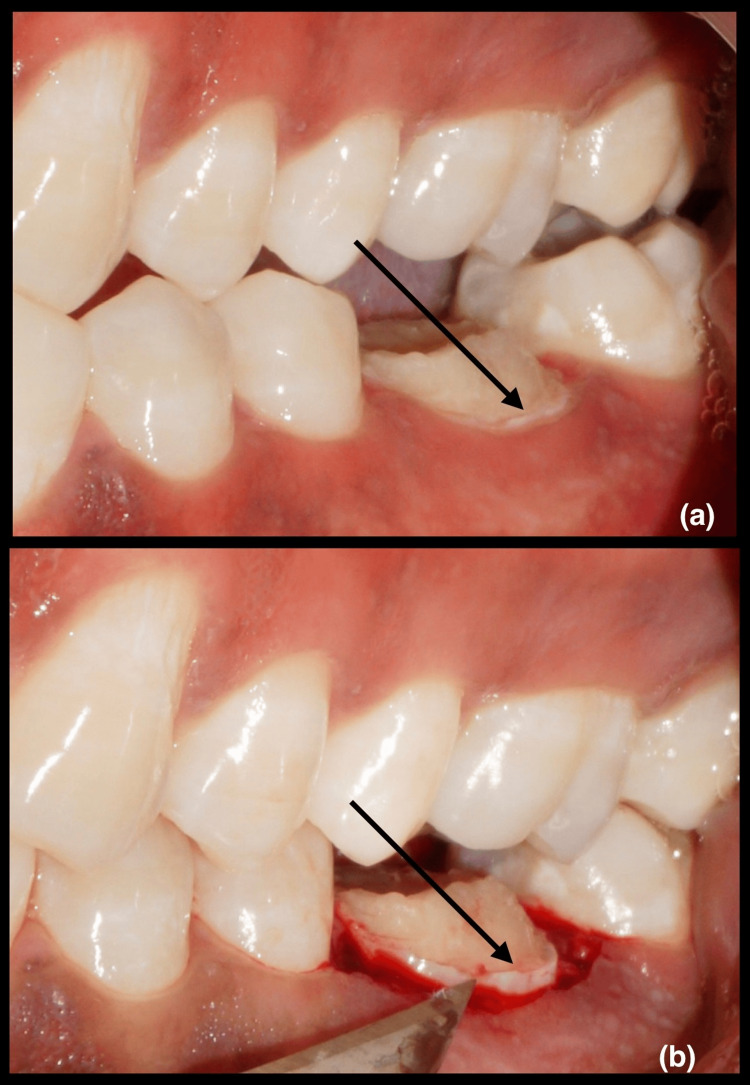
(a) Pre-operative image showing inadequate crown height in relation to 36; (b) intra-operative image showing crown lengthening procedure by gingivectomy

**Figure 2:**
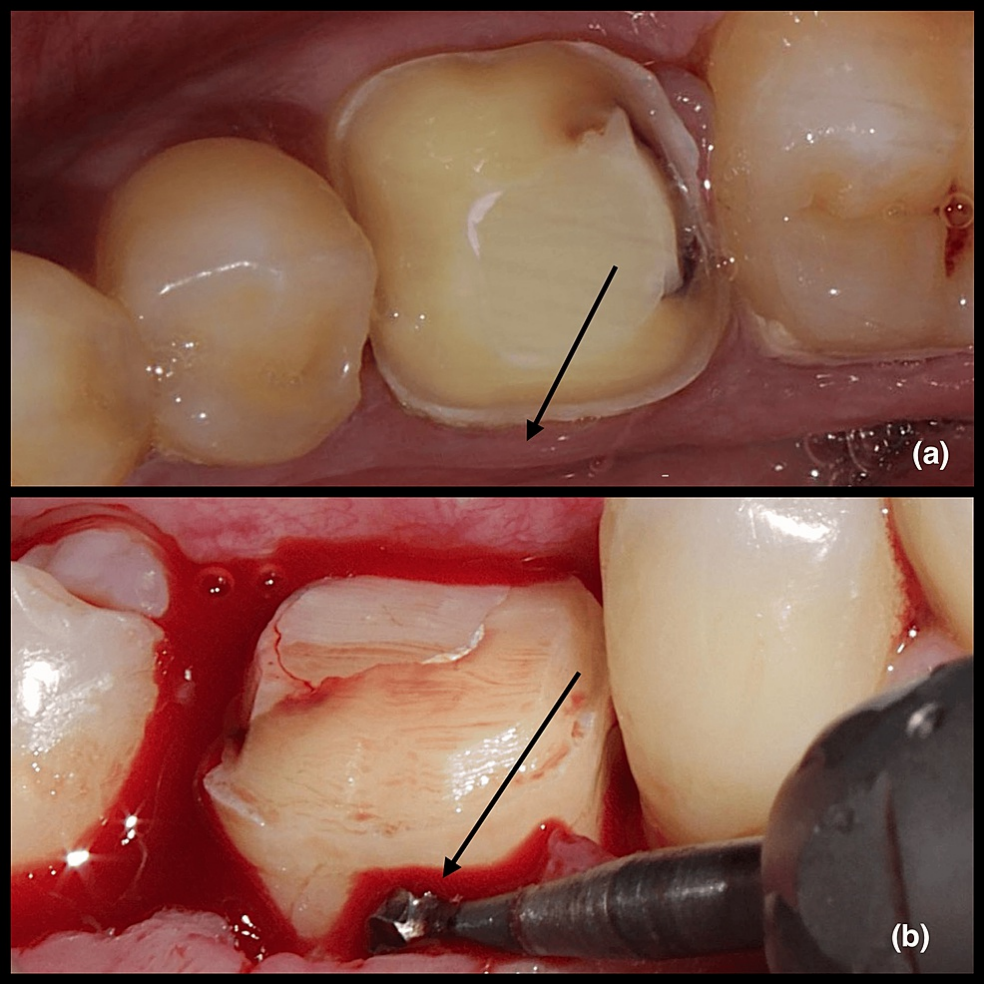
(a) Pre-operative image showing inadequate crown height in relation to 36; (b) intra-operative image showing crown lengthening procedure by apically displaced flap with osseous reduction

For group I patients, a gingivectomy was done (Figure 1) and for group II patients, an apically displaced flap with osseous reduction was done (Figure 2). For group II patients, sutures were removed 14 days following surgery. Both the group patients were prescribed to use 0.2% chlorhexidine gluconate mouth rinse twice daily for two weeks. Prosthetic treatment began no earlier than six weeks following surgery, and all patients continued with the maintenance program every three months. At three and six months after the surgery, the patient was anesthetized and measurements were obtained.

Statistical analysis

It was done with SPSS Software (Version 20.0, Armonk, NY, USA: IBM Corp). To assess variations in TT, AD, and NAD sites from baseline to three and six months, mean values were acquired for each site. Using these mean values, ANOVA was done. A p-value of <0.05 indicated that the result was statistically significant.

## Results

The mean age of participants in group I was 35.1±11.4 years and out of 30 subjects, 17 (61%) were males and 13 (39%) were females. In group II, the average age was 37.4±7.0 years with 15 (50%) males and 15 (50%) females (Table 1).

**Table 1:** Descriptive statistics Group I: gingivectomy; Group II: apically displaced flap with ostectomy. ^a^Chi-squared test ^b^Independent t-test

Variables	Study Groups		p-value
	Group I (n=30)	Group II (n=30)	
Male	17 (61%)	15 (50%)	0.899^a^
Female	13 (39%)	15 (50%)	
Age (years; mean±SD)	35.1±11.4	37.4±7.0	0.723^b^

Free gingival margin

The average distance from the fixed reference point in an acrylic stent to the FGM of TT, AD, and NAD sites of the tooth at baseline, three, and six months are elaborated in Table 2. At all the sites, there was a shift in FGM from baseline to three and six months. At three months, in group I, the FGM at TT, AD, and NAD sites were 3.5±0.16 mm, 3.07±0.9 mm, 2.8±0.21 mm, and in group II, it was 3.2±0.91 mm, 2.9±0.98 mm, 2.6±0.11 mm. At six months, group I exhibited 3.1±0.13 mm, 3.37±0.34 mm, 3.6±0.62 mm, and group II exhibited 3±0.31 mm, 3.4±0.51 mm, and 3.1±0.52 mm. There was no statistical difference between three and six months in terms of the position of FGM in both groups. When groups I and II were compared at three and six months, there was no statistical difference (p=0.126). On average at six months, in group I, there was 0.5 mm and 0.23 mm apical shift in FGM compared to group II (0.4 mm and 0.3 mm) at TT sites when compared to other sites (p<0.05) (Table 3).

**Table 2:** Clinical parameters acquired during baseline, six-month, and three-month examination at TT, AD, and NAD sites for both the study groups FGM, free gingival margin; RAL, relative attachment level; PD, probing depth; BL, bone level; BW, biologic width; TT, treated tooth sites; AD, adjacent tooth’s adjacent sites; NAD, adjacent tooth’s non-adjacent sites

Groups	Time period	FGM			PD			RAL			BL			BW		
		TT	AD	NAD	TT	AD	NAD	TT	AD	NAD	TT	AD	NAD	TT	AD	NAD
I	Baseline	4.4±0.18	4.43±0.4	4.1±0.19	2.3±0.85	2.46±0.42	2.98±0.48	7.28±1.53	7.56±1.28	7.66±1.38	9.43±1.65	9.78±1.46	9.56±1.44	2.45±0.3	2.03±.0.7	2.47±0.91
	3 months	7.9±0.21	7.5±0.48	6.9±0.31	3.3±0.74	2.42±0.85	2.64±071	10.3±1.245	10.2±1.97	10.8±1.6	12.45±1.21	12.4±1.38	12.7±1.45	1.14±0.45	1.54±0.98	1.43±0.61
	6 months	7.5±0.23	7.8±0.34	7.7±0.72	2.65±0.66	2.87±0.13	2.95±0.53	10.57±0.52	10.1±1.53	9.72±1.46	12.4±1.69	12.25±0.66	11.13±1.64	1.78±0.03	1.98±0.06	1.98±0.04
II	Baseline	4.6±0.28	4.5±0.25	4.2±0.19	2.5±0.65	2.56±0.52	2.58±0.78	7.17±1.43	7.24±1.18	7.16±1.18	9.33±1.75	9.67±1.56	9.46±1.34	2.23±0.2	2.24±0.11	2.34±0.08
	3 months	7.8±0.11	7.4±0.48	6.8±0.21	3.19±0.94	2.92±0.65	2.87±0.51	10.8±1.145	10.2±1.87	10.11±1.9	12.65±1.91	12.12±1.98	11.98±1.65	1.78±0.03	1.98±0.06	1.98±0.04
	6 months	7.6±0.3	7.9±0.41	7.3±0.42	2.74±0.76	2.59±0.53	2.38±0.53	10.67±0.32	10.04±1.43	9.98±1.56	12.9±1.89	12.45±0.76	11.43±1.54	2.17±0.09	2.09±0.12	2.15±0.07

**Table 3:** Mean changes from baseline to three months and six months at TT, AD, and NAD sites for both the study groups ^ˠ^p<0.05 from baseline ^*^p<0.05 from all other sites FGM, free gingival margin; RAL, relative attachment level; PD, probing depth; BL, bone level; BW, biologic width; TT, treated tooth sites; AD, adjacent tooth’s adjacent sites; NAD, adjacent tooth’s non-adjacent sites

Groups	Time period	FGM			PD			RAL			BL			BW		
		TT	AD	NAD	TT	AD	NAD	TT	AD	NAD	TT	AD	NAD	TT	AD	NAD
I	3 months	3.5±0.16	3.07±0.9	2.8±0.21	1±0.54	-0.04±0.1	-0.34±061	3.02*±1.3	2.64±1.05	3.14±1.01	3.02±1.31	2.62±1.28	3.14±1.55	-1.31^ˠ^±0.55	-0.49^ˠ^±0.78	-1.04^ˠ^±0.61
	6 months	3.1*±0.13	3.37±0.34	3.6±0.62	0.35±0.01	0.41±0.23	-0.03±0.43	3.29*±0.12	2.54±1.01	1.95±1.06	2.97*±1.09	2.47±0.16	1.57±1.74	-0.67^ˠ^±0.13	-0.05^ˠ^±0.06	-0.46^ˠ^±0.02
II	3 months	3.2±0.91	2.9±0.98	2.6±0.11	0.69±0.12	0.36±0.14	0.29±0.21	3.63*±1.11	2.96±1.07	2.95±1.2	3.32±1.721	2.45±1.82	2.52±1.75	-0.45^ˠ^±0.13	-0.26^ˠ^±0.16	-0.36^ˠ^±0.03
	6 months	3*±0.31	3.4±0.51	3.1±0.52	0.24±0.51	0.03±0.43	-0.2±0.03	3.5*±0.12	2.8±1.21	2.38±1.46	3.57*±1.2	2.78±0.66	1.97*±1.41	-0.06^ˠ^±0.19	-0.15^ˠ^±0.22	-0.19^ˠ^±0.08

Probing depth

Table 2 lists the mean PDs of TT, AD, and NAD sites of the tooth at baseline, three, and six months. The PD increased from baseline to three months at all sites, then dropped by six months, but the differences were statistically insignificant (p>0.05). At any site and between both the groups, the mean probing at baseline, three, and six months did not differ statistically (p>0.05) (Table 3).

Relative attachment level

Table 2 lists the mean RALs of TT, AD, and NAD sites of the tooth at baseline, three, and six months. At all sites in both groups, from baseline to three and six months, the base of the sulcus had an apical shift (p<0.05). TT sites in both groups demonstrated statistical significance when compared to other sites at three and six months (p<0.05). During three and six months, the attachment loss for TT sites was higher than for NAD and AD sites (p<0.05). At any given time, there was no discernible difference between the two groups (p=0.897) (Table 3).

Bone level

The mean BL of TT, AD, and NAD sites of the tooth at baseline, three, and six months are described in Table 2. TT sites in group I at six months exhibited a statistically significant difference in BL when compared to other sites and in group II, both TT and NAD sites exhibited a statistically significant difference in BL when compared to AD sites (p<0.05). Also, BL was statistically significant when both groups were compared at a given time with a specific site (p<0.05) (Table 3).

Biologic width

The average BW of TT, AD, and NAD sites of the tooth at baseline, three, and six months are described in Table 2. The BW at all sites in both groups was lesser at three and six months than at baseline (Table 3). When BW was compared between the two groups at three and six months, group II showed better reestablishment of BW at any given time period and was statistically significant (p<0.05) (Table 3).

## Discussion

Many failures in complex periodontal-prosthetic situations have been linked to the restorative margins violating biological width [14]. This clinical study was done to assess the long-term periodontal changes after two different crown lengthening (gingivectomy and apically positioned flap with ostectomy) procedures. This study was mainly done to witness the procedure, which restores maximum BW.

This human clinical investigation illustrated at least a 3 mm gain in crown structure post-gingivectomy and apically positioned flap with ostectomy procedures at three and six months. The BW was substantially reduced from baseline at all the examined study sites after both the crown lengthening procedures. When teeth treated with apically positioned flap with ostectomy and teeth treated with gingivectomy were compared postoperatively, BW variations were between 0.05 mm and 1.31 mm and 0.06 mm to 0.45 mm, respectively. These results agree with those of earlier research. Lanning et al. showed that six months after an apically positioned flap with ostectomy, the BW at treated sites returned to its initial vertical dimension [15]. Similarly, Ganji et al. reported that there was more increase in BW in the sixth month after an apically positioned flap with ostectomy compared to the gingivectomy procedure. Additionally, it was observed in this study that at the end of the sixth month, the BW at all sites nearly reached the baseline measurements in the group treated with an apically positioned flap with ostectomy.

Gingiva also prefers to be close to the cementoenamel junction. The dynamic action of the gingiva, which always seems to revert to its pre-operative architectural form and restore the BW, has been highlighted in a previous study [16]. In addition, Carnevale et al. have proposed that following a surgical crown lengthening procedure, bone resorption produces supracrestal tooth structure, which allows connective tissue to adhere and restore the BW [17]. However, sufficient information has not been provided in the literature regarding the dimension of the post-surgical BW and BL modifications and their stability over time following gingivectomy with and without osseous reduction. Furthermore, the changes in the AD following gingivectomy with and without osseous reduction were not studied. In this context, the rationale of this study was to assess the periodontal tissue changes in the treated and AD following crown lengthening procedures with and without bone reduction.

In addition, it was noted that there was no change in the baseline and sixth-month examination values for either group, with the PD values generally returning to their presurgical values. However, an increase in RAL measurements was found at the completion of the study compared to the baseline measurements in both groups. Furthermore, this increase in attachment level was slightly more among the group treated with apically positioned flap with ostectomy. These results could point to the periodontium's propensity to regenerate a physiological supracrestal gingival unit [18]. These results were consistent with the Lindhe et al. [19]. They suggested that the gingival margin's position was moved apically during active periodontal therapy, but this displacement was slightly balanced by coronal regrowth that occurred during the postoperative maintenance care phase.

Additionally, the current study proved that among the group treated with an ostectomy and apically positioned flap, the free marginal gingiva showed a greater propensity to grow in a coronal direction at the six-month follow-up. This finding is contrary to Ganji et al. as they reported that the gingivectomy procedure showed a distinct tendency to grow the marginal periodontal tissues in a coronal direction [13]. Crown lengthening procedures carried out for restorative purposes have been the subject of a recent systematic review, which included five clinical trials. These studies, however, were deemed to be highly biased, and none of them provided a clear statement about whether the postoperative outcome was sufficient for the intended restorative purposes or had a follow-up period longer than six months. Moreover, no trials comparing surgical techniques were found [20]. A recent meta-analysis evaluated how clinical parameters at AD and NAD were affected by periodontal crown lengthening surgery in comparison to treated sites and observed significant alterations in treated, AD, and NAD were made possible by the surgery. When comparing treated sites to AD and NAD, there were more alterations in PD and CAL [21].

Limitations

This study failed to assess the tissue biotype, healing characteristics unique to each patient, BW reformation, amount of bone reduction, the time frame of restoration, plaque control following surgery, localized site, and the location of the flap margin following surgery as these factors could influence the treatment outcome. All these factors could be the confounding factors of the present study. 

## Conclusions

The present study data suggests that following surgical crown lengthening, the BL was shifted apically and allowed for the reestablishment of BW. At six months of follow-up, the apically positioned flap with ostectomy was superior in restoring the BW compared to gingivectomy. In addition, clinical parameter changes after crown lengthening surgery were more pronounced at treated sites. However, clinical alterations can also happen at NAD and AD, which can affect the clinical outcome. Also, the level of existing bone and width of the attached gingiva need to be considered before deciding the procedure.
